# Supplementary material for: Staphylococcus schweitzeri—An Emerging One Health Pathogen?
Source: Microorganisms. 2022 Apr 2;10(4):770. doi: 10.3390/microorganisms10040770 (PMC9026344; doi:10.3390/microorganisms10040770)
Supplement: Supplementary file 1 [file microorganisms-10-00770-s001.zip › Supplementary Figure 1.pdf]

```
DQ507381.1      -----------------------------------ATGACAGAATACTTATTAAGTGCTG 25
MN166565.1      ATGAAAATTAACTAAAAAAGAAAGAGGTGTTAGTTATGACAGAATACTTTTTAAGTGCTG 60
KJ748637.1      -----------------------------------ATGACAGAATACTTTTTAAGTGCTG 25
KJ748638.1      -----------------------------------ATGACAGAATACTTTTTAAGTGCTG 25
KJ748639.1      -----------------------------------ATGACAGAATACTTTTTAAGTGCTG 25
OE998560.1      -----------------------------------ATGACAGAATACTTTTTAAGTGCTG 25
                                                   ************** **********

DQ507381.1      GCATATGTATGGCAATTGTTTCAATATTACTTATAGGGATGGCTATCAGTAATGTTTCGA 85
MN166565.1      GCATATTTACGGCAATTGTTTCATTAGTACTTGTAGTGATGGCTATAAGTAAATTTTCTA 120
KJ748637.1      GCATATTTACGGCAATTGTTTCATTAGTACTTATAGTGAAGGCTATAAGTAAACTTTCTC 85
KJ748638.1      GCATATTTACGGCAATAGTTTCATTAGTACTTATAGTGATGGCTATAAGTAAACTTTCTC 85
KJ748639.1      GCATATTTACGGCAATAGTTTCATTAGTACTTATAGTGATGGCTATAAGTAAACTTTCTC 85
OE998560.1      GCATATTTACGGCAATAGTTTCATTAGTACTTATAGTGATGGCTATAAGTAAATTTTCTC 85
                ****** ** ****** ****** ** ***** *** ** ****** *****  ****

DQ507381.1      AAGAACAATACGCAAAGAGGTTTTTCTTTTTCGCTACTAGTTGCTTAGTGTTAACTTTAG 145
MN166565.1      AAAGGCAAACAGCGAAAAGATTTTTATTTTTCTCTATTAGTTGCTTAGTGTTAACATTAG 180
KJ748637.1      AAAGGCATACAATGAAAAGATTTTTATTTTTATCTACTAGTTGCTTAGTGTTAACATTAG 145
KJ748638.1      AAAGGCAAACCGCGAAAAAATTTTTATTTTTCTCTACTAGTTGCTTATTGTTAACATTAG 145
KJ748639.1      AAAGGCAAACCGCGAAAAGATTTTTATTTTTCTCTACTAGTTGCTTAGTGTTAACATTAG 145
OE998560.1      AAAGGCAAACCGCGAAAAGATTTTTATTTTTCTCTACTAGTTGCTTAGTGTTAACATTAG 145
                **   **       ** *  ***** *****  *** ********** ******* ****

DQ507381.1      TTGTAGCTTCAAGTCTAAGTAGCTCAGCAAATGCATCACAAACAGATAATGGCGTAAATA 205
MN166565.1      TTGTAGTTTCTAATTTTAATCAATCTGCAGGAGCATCCCAACTAACTGATGAAGAG---- 236
KJ748637.1      TTGTAGTTTCTAACTTTAATCAATCTGCAGGAGCTGCTCAACTAGCTGATGAAGAT---- 201
KJ748638.1      TTGTAATTTCTAACTTTAATCAATCTGCAGGAGCTGCCAAACTAGCTGATGAAGAT---- 201
KJ748639.1      TTGTAATTTCTAATTTTAATCAAACTGCTGGAGCTTCCCAACTAACTGATGAAGAT---- 201
OE998560.1      TTGTAATTTCTAATTTTAATCAAACTGCTGGAGCTTCCCAACTAACTGATGAAGAT---- 201
                *****  *** *   * * *    * **    **  *  **  *  * ***  *

DQ507381.1      GAAGTGGTTCTGAAGATCCAACAGTATATAGTGCAACTTCAACTAAAAAATTACATAAAG 265
MN166565.1      -----GGAAATAGCAATGCAACTGAATACAGTAAATCAGCAACGACTAAATTACATAAAG 291
KJ748637.1      -----GGAAATAGCAATACAACTGAATACAGCAAATCAGCAACGACTAAATTACATAAAG 256
KJ748638.1      -----GGAAATAGCAATACAACTGAATATAGCAAGTCAGCAACGACTAAATTACATAAAG 256
KJ748639.1      -----GGAAATAGCAATGCAACTGAATATAGCAAGTCAGCAACGACTAAATTACATAAAG 256
OE998560.1      -----GGAAATAGCAATGCAACTGAATATAGCAAGTCAGCAACGACTAAATTACATAAAG 256
                     **   *    ** **** * *** **     *  **** *  *************

DQ507381.1      AACCTGCGACATTAATTAAAGCGATTGATGGTGATACTGTTAAATTAATGTACAAAGGTC 325
MN166565.1      AACCTGCGACATTAATTAAAGCGATCGATGGTGATACGGTTAAGTTAATGTATAAAGGAA 351
KJ748637.1      AGCCTGCGACATTAATCAAAGCAATTGATGGTGATACTGTTAAGTTAATGTACAAAGGAA 316
KJ748638.1      AGCCTGCGACATTAATCAAAGCAATTGATGGTGATACTGTTAAGTTAATGTACAAAGGAA 316
KJ748639.1      AGCCTGCGACATTAATCAAAGCAATTGATGGTGATACTGTTAAGTTAATGTACAAAGGAA 316
OE998560.1      AGCCTGCGACATTAATCAAAGCAATTGATGGTGATACTGTGAAGTTAATGTACAAAGGAC 316
                * ************** ***** ** *********** ** ** ******** *****

DQ507381.1      AACCAATGACATTCAGACTATTATTGGTTGATACACCTGAAACAAAGCATCCTAAAAAAG 385
MN166565.1      AGGCAATGACATTTAGATTGTTATTAATTGATACGCCTGAAACGAAACATCCTAAAAAAG 411
KJ748637.1      AACCAATGACATTTAGATTGTTATTAATTGATACGCCCGAAACGAAACATCCTAAAAAAG 376
KJ748638.1      AACCAATGACATTTAGATTGTTATTAATTGATACGCCCGAAACGAAACATCCTAAAAAAG 376
KJ748639.1      AACCAATGACATTTAGATTGTTATTAATTGATACGCCCGAAACGAAACATCCTAAAAAAG 376
OE998560.1      AACCAATGACATTTAGATTGTTATTAATTGATACGCCCGAAACGAAACATCCTAAAAAAG 376
                *  ********** *** * *****  ******* ** ***** ** *************

DQ507381.1      GTGTAGAGAAATATGGTCCTGAAGCAAGTGCATTTACGAAAAAGATGGTAGAAAATGCAA 445
MN166565.1      GCGTTGAAAAATATGGCCCAGAAGCAAGTGCATTTACGAAGAATATGGTAGAGAATGCTA 471
KJ748637.1      GCGTTGAAAAATACGGACCAGAAGCAAGCGCATTTACGAAAAAAATGGTAGAGAATGCTA 436
KJ748638.1      GCGTTGAAAAATACGGACCAGAAGCAAGCGCGTATACGAAAAAAATGGTAGAGAATGCTA 436
KJ748639.1      GCGTTGAAAAATACGGGCCAGAAGCAAGCGCGTTTACGAAAAAAATGGTAGAGAATGCTA 436
OE998560.1      GCGTTGAAAAATATGGACCTGAAGCAAGTGCGTTTACAAAAAAAATGGTAGAGAATGCTA 436
                * ** ** ***** ** ** ******** ** * *** ** ** ******** ***** *

DQ507381.1      AGAAAATTGAAGTCGAGTTTGACAAAGGTCAAAGAACTGATAAATATGGACGTGGCTTAG 505
MN166565.1      AGAAAATAGAAGTGGAATTTGACAAAGGTCAAAAGACAGATAAATATGGACGTGGCTTAG 531
KJ748637.1      AAAAAATTGAAGTTGAATTTGACAAAGGACAAAAAACTGATAAATATGGACGTGGATTAG 496
KJ748638.1      AGAAAATTGAAGTTGAATTTGACAAAGGACAAAAAACTGATAAATATGGACGTGGATTAG 496
KJ748639.1      AGAAAATTGAAGTTGAATTTGACAAAGGACAAAAAACTGATAAATATGGACGTGGATTAG 496
OE998560.1      AGAAAATTGAAGTTGAATTTGACAAAGGACAAAAGACAGATAAATATGGACGTGGATTAG 496
                * ***** ***** ** *********** ****  ** **************** ****

DQ507381.1      CGTATATTTATGCTGATGGAAAAATGGTAAACGAAGCTTTAGTTCGTCAAGGCTTGGCTA 565
MN166565.1      CTTATATCTATGCTGATGGTAAGATGGTTAACCAAGCTTTAGTGCGTCAAGGTCTAGCAA 591
KJ748637.1      CTTATATTTATGCAGATGGCAAAATGGTTAATCAAGCGTTAGTACGTCAAGGATTAGCAA 556
KJ748638.1      CTTATATTTATGCAGATGGTAAAATGGTTAACCAAGCATTAGTGCGTCAAGGATTAGCAA 556
KJ748639.1      CTTATATTTATGCAGATGGCAAAATGGTTAATCAAGCGTTAGTACGTCAAGGATTAGCAA 556
OE998560.1      CTTATATTTATGCAGATGGTAAAATGGTTAACCAAGCATTAGTGCGTCAAGGATTAGCAA 556
                * ***** ***** ***** ** ***** **  **** ***** ********  * ** *

DQ507381.1      AAGTTGCTTATGTTTATAAACCTAACAATACACATGAACAACTTTTAAGAAAAAGTGAAG 625
MN166565.1      AAGTTGCGTATGTTTATAAACCTAATAACACACATGAACAACTATTAAGAAAAAGTGAAG 651
KJ748637.1      AAGTTGCATATGTATATAAACCTAATAATACACACGAACAACTATTAAGGAAAAGTGAAG 616
KJ748638.1      AAGTTGCATATGTATATAAACCTAATAATACACATGAACAACTACTAAGAAAAAGTGAAG 616
KJ748639.1      AAGTTGCATATGTATATAAACCTAATAATACACATGAACAACTATTAAGGAAAAGTGAAG 616
OE998560.1      AAGTTGCATATGTATATAAACCTAATAATACACATGAACAACTACTAAGAAAAAGTGAAG 616
                ******* ***** *********** ** ***** ********  **** **********

DQ507381.1      CACAAGCGAAAAAAGAGAAATTAAATATTT------------------------------ 655
MN166565.1      CACAAGCGAAAAAAGAACATTTAAATATTTGGAGTGAAGGTAACGCAGAATAAAGTTACT 711
KJ748637.1      CACAAGCGAAAAAAGAACATCTAAATATTTGGAGTGAAGGTAACGCAGAATAAAGTTACT 676
KJ748638.1      CAAAAGCGAAAAAAGAACATCTAAATATTTGGAGTGAAGGTAACGCAAAATAAAGTTACT 676
KJ748639.1      CACAAGCGAAAAAAGAACATCTAAATATTTGGAGTGAAGGTAACGCAGAATAA------- 669
OE998560.1      CACAAGCGAAAAAAGAACATTTAAATATTTGGAGTGAAGGTGACGCAGAATAA------- 669
                ** *************  *  *********

DQ507381.1      ------------------------------------------------------       655
MN166565.1      GACATTTAACATAAAGTGCCACTGCTGCTAGTGGCACTTTTATAATTTTTAGAT       765
KJ748637.1      G-----------------------------------------------------       677
KJ748638.1      G-----------------------------------------------------       677
KJ748639.1      ------------------------------------------------------       669
OE998560.1      ------------------------------------------------------       669
```

Supplementary Figure S1: Alignment of *nuc* sequences. Nuc sequences (GeneBank accession numbers in brackets) of *Staphylococcus aureus* (DQ507381.1), *Staphylococcus argenteus* (MN166565.1), *Staphylococcus schweitzeri* (KJ748637.1, KJ748638.1, KJ748639.1) and *Staphylococcus roterodami* (OE998560.1) were aligned using the Clustal Omega multiple alignment tool (https://www.ebi.ac.uk/Tools/msa/clustalo/). The primer binding site of the nuc-PCR suggested by Brakstad et al. (J. Clin. Microbiol. 1992) are underlined and shaded in grey.
