# Supplementary material for: Staphylococcus schweitzeri—An Emerging One Health Pathogen?
Source: Microorganisms. 2022 Apr 2;10(4):770. doi: 10.3390/microorganisms10040770 (PMC9026344; doi:10.3390/microorganisms10040770)
Supplement: Supplementary file 1 [file microorganisms-10-00770-s001.zip › Supplementary Figure 2.pdf]

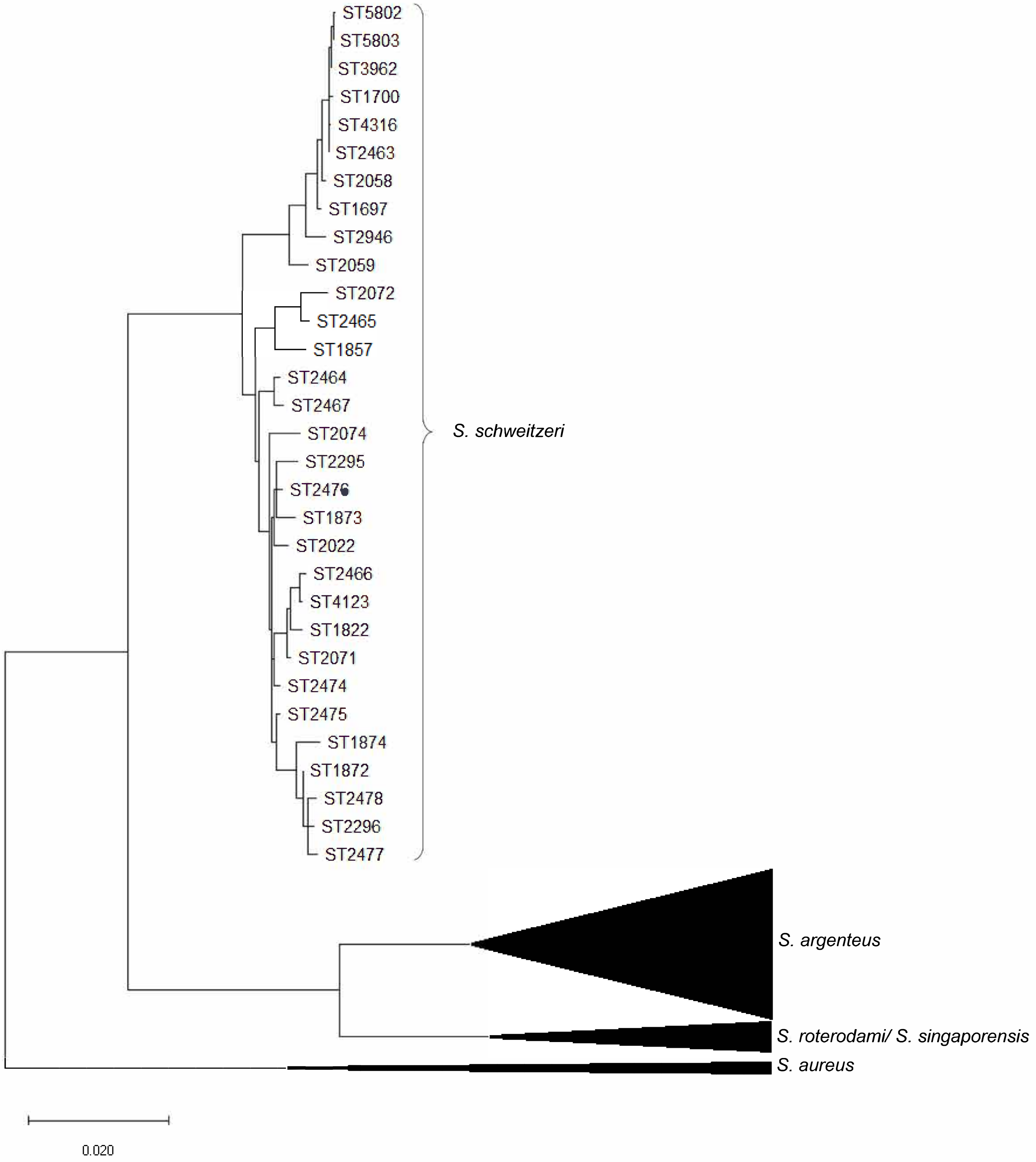

Supplementary Figure S2: Phylogenetic tree of members of the *Staphylococcus aureus*-related complex. The concatenated sequences of the seven alleles included in the multilocus sequence typing (MLST) scheme were used to construct phylogenetic tree based on the the Maximum Likelihood method. The sequences were obtained from the PubMLST database (https://pubmlst.org/). The tree is rooted to the *S. aureus* sequence types (ST) ST5, ST22, ST30, ST45 and ST398 (collapsed).
